# Acceptability and Usability of the Mobile Digital Health App NoObesity for Families and Health Care Professionals: Protocol for a Feasibility Study

**DOI:** 10.2196/18068

**Published:** 2020-07-22

**Authors:** Edward Meinert, Em Rahman, Alison Potter, Wendy Lawrence, Michelle Van Velthoven

**Affiliations:** 1 Digitally Enabled PrevenTative Health (DEPTH) Research Group Department of Paediatrics Univeristy of Oxford Oxford United Kingdom; 2 Health Education England Southhampton United Kingdom; 3 Medical Research Council Lifecourse Epidemiology Unit University of Southampton Southampton United Kingdom; 4 National Institute for Health Research Southampton Biomedical Research Centre University Hospital Southampton NHS Foundation Trust Southampton United Kingdom

**Keywords:** mHealth, mobile health, digital health, digital technology, weight loss, obesity, overweight, child health, cell phone, telecommunication

## Abstract

**Background:**

Almost a quarter or more than a fifth of children in the United Kingdom are overweight or obese by the time they start school. The UK Department of Health and Social Care’s national policy for combating childhood obesity has critical outcomes centered on sugar and caloric consumption reduction. Health Education England has developed two digital apps for families with children up to 15 years and for their associated health care professionals to provide a digital learning resource and tool aimed at encouraging healthy lifestyles to prevent obesity.

**Objective:**

This feasibility study assesses the usability and acceptability of Health Education England’s NoObesity app for undertaking activities to improve families’ diet and physical activity. The purpose of the study is to evaluate the app’s influence on self-efficacy and goal setting and to determine what can be learnt to improve its design for future studies, if there is evidence of adoption and sustainability.

**Methods:**

The study population will include 20 to 40 families and their linked health care professionals. Considering issues related to digital access associated with socioeconomic status and the impact on information technology use, study recruitment will be regionally focused in a low socioeconomic status area. The study will last for 9 months (3-month intervention period and 6-month follow-up). The evaluations of feasibility, acceptability, and usability will be conducted using the following scales and theoretical frameworks: (1) system usability scale; (2) Reach Effectiveness Adoption Implementation Maintenance framework; (3) Bandura model of health promotion; and (4) Nonadoption, Abandonment, and Challenges to the Scale-up, Spread, and Suitability framework. App use will be captured and quantitatively analyzed for net use patterns (eg, number of screens viewed, number of logins, cumulative minutes using the app, number of plans made, and number of times goals met) and to triangulate qualitative feedback from study participants.

**Results:**

This study was funded in March 2019 by Health Education England and received University of Oxford Medical Sciences Interdivisional Research Ethics Committee approval on January 31, 2020 (R62092/RE001). At manuscript submission, study recruitment is pending, and expected results will be published in 2021.

**Conclusions:**

This study will provide evidence on the NoObesity app’s influence on self-efficacy and goal-setting and determine what can be learnt to improve its design for future studies, if there is evidence of adoption and sustainability.

**International Registered Report Identifier (IRRID):**

PRR1-10.2196/18068

## Introduction

### Background

Obesity is a rising concern globally. In the United Kingdom, it is projected that by 2030, 41% to 48% of men and 35% to 43% of women will be obese, and almost a quarter or more than a fifth of children are overweight or obese by the time they start school [1]. It is estimated that obesity-related conditions are currently costing the National Health Service £6.1 billion (US $7.3 billion; £1=US $1.23) per year, with a cost to society of these conditions estimated at £27 billion (US $33.3 billion) per year [2-4]. The UK Department of Health and Social Care updated the national policy for combating childhood obesity in 2018, with critical outcomes centered on sugar and calorie consumption reduction [2].

The rapid development of technology has quickly led to a growing market for various devices and mobile digital software claiming to aid with weight loss, with 102.4 million products sold in 2016, and sales expected to continue to rise [5]. The effectiveness of these technologies has been subject to many studies [6-10], with some results suggesting that they can benefit weight loss temporarily. However, sustained weight loss is often unsuccessful [8,9]. Even when evidence on these interventions demonstrates a relevant weight loss or reduction in weight gain, if this benefit only lasts for a short period, it dramatically reduces the usefulness of these technologies [8,9]. Digital mobile technology for weight loss could be a novelty that wears off over time, rather than an intervention for sustained lifestyle change that can be maintained over the long term. While these technologies have high potential, the limitations noted require further analysis to see how the approaches can be used to make a lasting impact on positive lifestyle change.

Health Education England developed the NoObesity app as a collaborative initiative between the Universities of Bournemouth and Southampton in 2017-2018. This work centered on the development of (1) a family-focused app to enable families to set health goals, identify barriers, and develop strategies to overcome them and (2) a professional-focused app to help health care professionals provide tailored advice to families, provide information on how to handle common objections, and assist families with their lifestyle objectives. The project completed development and user testing on Apple and Android platforms by producing, testing, and finalizing the functionality and structure of the app and also writing, testing, and finalizing the content of the app. User testing consisted of focus groups conducted with professionals and parents or care givers [11]. This feasibility study aims to gather data on the usability and acceptability of the NoObesity app for undertaking activities involving improving family diet, physical activity, and weight.

### Solution Overview

NoObesity involves two apps (NoObesity Professional and NoObesity Family) to support the prevention and management of childhood obesity targeting professionals and families. The apps focus on developing the workforce to support families around childhood obesity and enabling families to set behavioral goals to support their own health and wellbeing.

The apps bring both workforce development and service delivery together, which is the result of collaboration that led to the development of this project.

#### NoObesity Professional App

**Figure 1:**
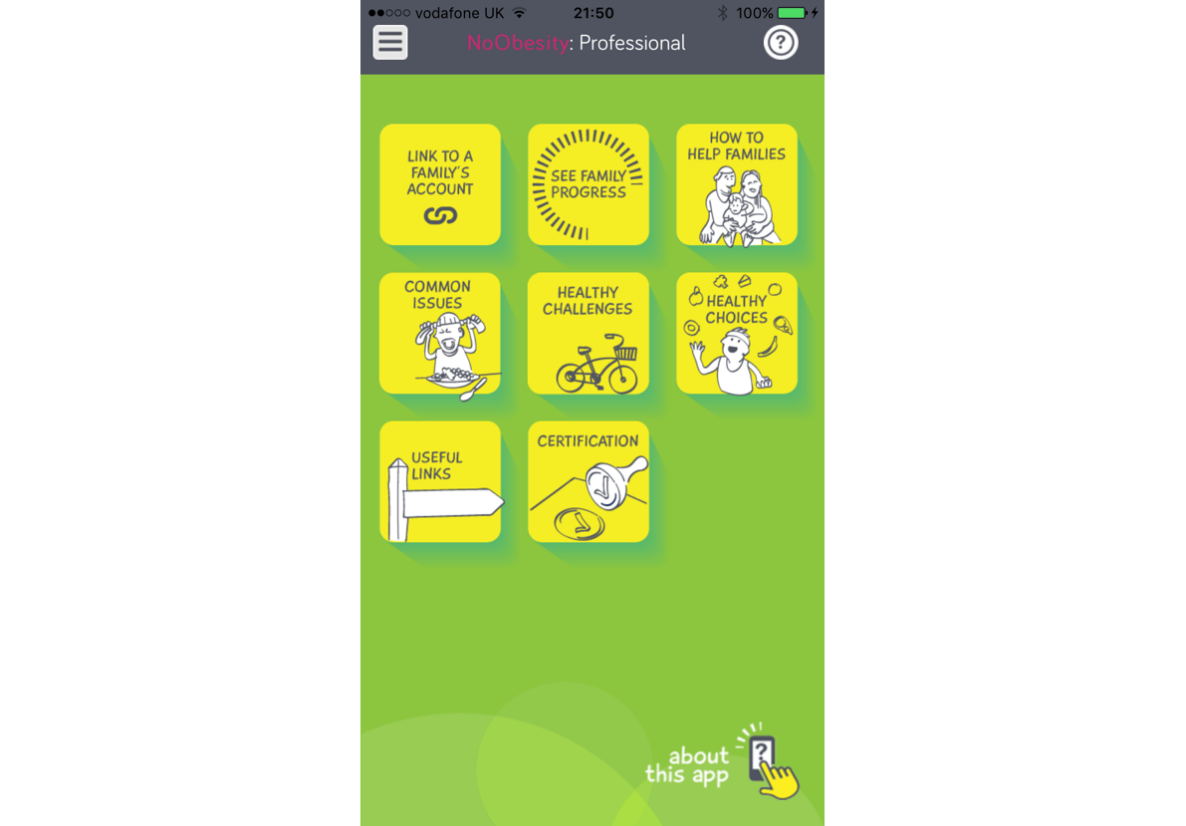
Main screen of the NoObesity Professional app.

The NoObesity Professional app has functionality that supports knowledge and skill development of the workforce centered on weight management. Figure 1 outlines the functions in the NoObesity Professional app, and Table 1 outlines the purposes and outcomes.

#### NoObesity Family App

**Figure 2:**
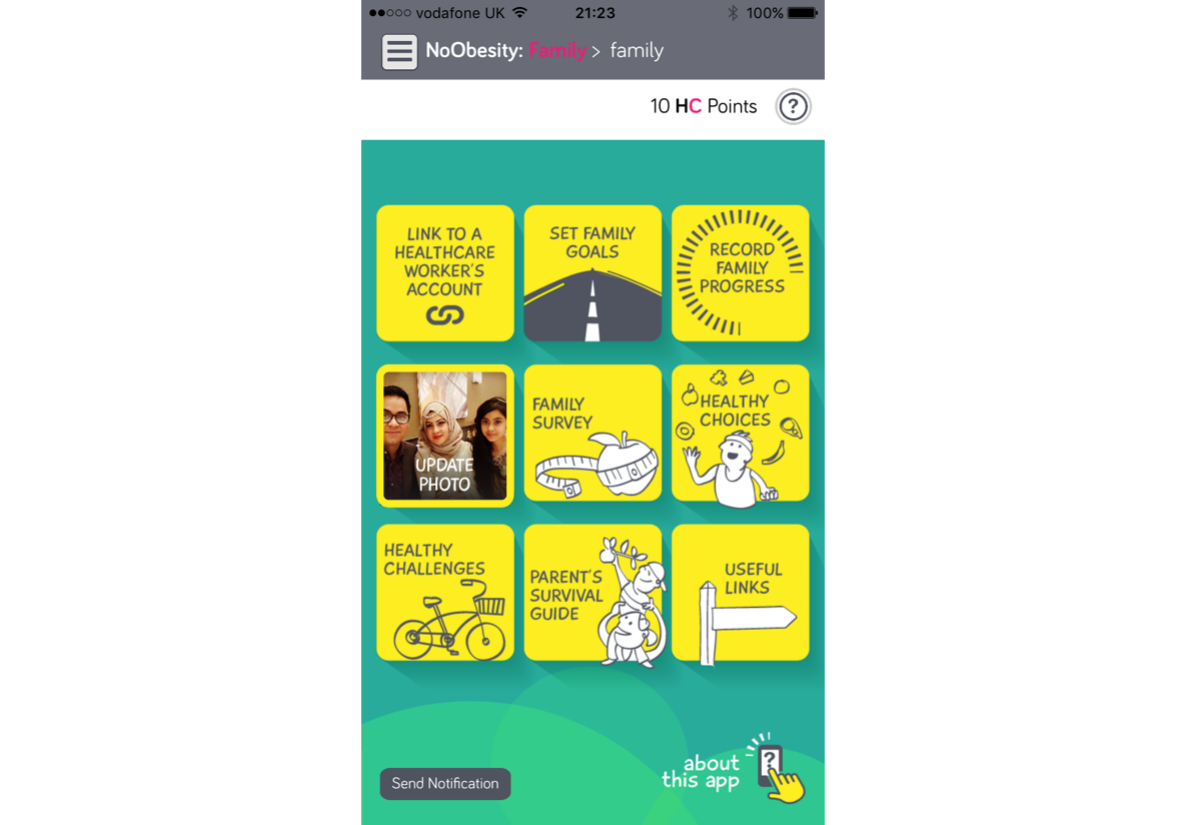
Main screen of the NoObesity Family app.

The NoObesity Family app has functionality that enables families to set behavioral goals to support their own health and wellbeing. These are highlighted in Figure 2. Table 2 outlines the purposes and outcomes of the family app use cases.

**Table 1:** NoObesity Professional app functional overview.

App function	Purpose	Outcome
How to help families	This function enables the professional to follow a family (through a story), where the family comes into contact with a number of different health care workers. The tasks for the professional are as follows: Read the section of the story. Score whether the interaction was good. Reflect on whether there were any missed opportunities to talk about health and wellbeing with the family. The app then provides the professional with information on how interactions with the family can be improved for their own practice.	Professionals will have an increased awareness of the potential opportunities that could be missed in supporting families around health and wellbeing. Professionals will be enabled to reflect on their own practice when coming into contact with families. Professionals will see (read) how a Making Every Contact Count (MECC) approach can be used for their own practice in supporting families.
Common issues	This function provides some of the common issues professionals face when supporting families around healthy eating, diet, and activity. This function offers professionals with some solutions on how they can overcome the common issues and challenges they face. The solutions are developed with MECC principles, which allow professionals to use MECC skills in supporting families.	Professionals are able to learn about solutions they can use to overcome issues and challenges they are presented with. Professionals are enabled to practice MECC skills, as they support families to overcome issues and challenges.
Healthy challenges & healthy choices	These two functions are developed in the form of games within the app that professionals can play as an approach to learning in a fun way about what makes a healthy diet and what level of physical activity is required to burn off calories.	Professionals will gain knowledge of the Eatwell Plate and physical activity to support families.
Useful links	This function provides links to websites for professionals to access for furthering their own learning and Continuing Professional Development.	Professionals will learn about the resources available on specific aspects of health and wellbeing in order to be better informed on the topic and possible signposting.
Certification	This function encourages professionals to do all of the above as they gain bronze, silver, and gold awards on further exploring the content of the app.	Professionals have used the app to develop their knowledge and skills in supporting the prevention and management of childhood obesity with the families they support.

**Table 2:** NoObesity Family app functional overview.

App function	Purpose	Outcome
Set family goals	This function enables families to set behavioral goals to support them in their health and wellbeing. This function takes them through a Specific, Measurable, Action-oriented, Realistic, Timed, Evaluated, Reviewed (SMARTER) process to make plans and set goals.	Families set goals using a SMARTER process that is part of the MECC program.
Record family progress	This function allows families to record how they have been progressing with family goals and enables them to evaluate and review their goals if they have not progressed as expected.	Families are able to record their progress and review their goals.
Update photo	This function is in place for families to be able to upload pictures of themselves doing healthy things. This could be a meal they have cooked, pictures of them on a health walk, etc.	This is used as an engagement tool allowing families to upload photos of things they have done as a way of encouraging them to come back to the app.
Family survey	This function supports families to take stock of where they are around a number of health-related areas. The family survey includes the following questions: How much water do you drink? How much fruit do you eat? How many vegetables do you eat? How much physical activity do you do? How much sleep do you have? How much screen time do you have? How often do you brush your teeth? How happy do you feel? What is your body size? These questions can be completed by more than one person, so you could end up with a health survey for all members of the family. Once the survey is completed, the app will prompt families to think about setting a goal around one of the areas of the health survey (if they have scored low on it).	It enables collection of baseline data to allow for comparison of change.
Healthy choices & healthy challenges	This function is the same as in the Professional app.	It enables families to learn about the Eatwell Plate and about physical activity.
Parent’s survival guide	This function supports families in thinking about how they can overcome the challenges that they face when supporting their children around healthy eating, etc.	Families gain solutions on how they could approach issues and challenges using a MECC approach.
Useful links	This function is the same as in the Professional app.	It enables families to learn about other resources available to support their health and wellbeing.

#### Linking the Apps

The two apps can be linked using a QR code, which is led by the family who may wish to share the goals and progress they have made with a health care worker. For example, this could be during a general practitioner consultation or a visit to the local children’s center. The professional will only see the family’s survey results, goals, and progress. This will allow the professional to better tailor the support they provide to the family.

### Research Questions

**Figure 3:**
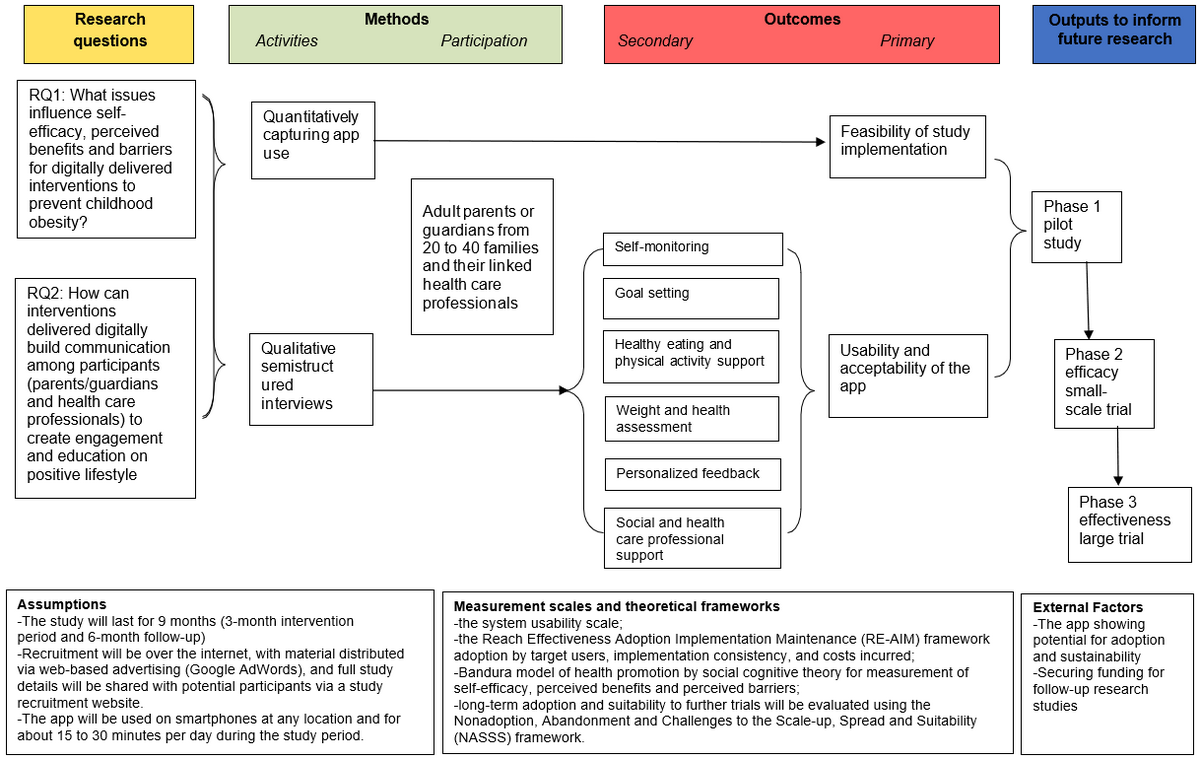
Study logic model.

The purpose of this study is to investigate the following research questions (Figure 3): (1) What are the issues impacting self-efficacy, perceived benefits, and barriers for digitally delivered interventions to prevent childhood obesity (specifically examining learning and associated actions with regard to diet and physical activity)? (2) How can interventions delivered digitally (eg, NoObesity app) build communication among participants (parents or guardians and health care professionals) to create engagement and education on positive lifestyle habits (parents or guardians and health care professionals)?

## Methods

### Design

This investigation will take the form of a feasibility study using mixed methods. To address the study research questions, quantitative monitoring of app users will be supplemented by qualitative interviews with parents or guardians and health care professionals (Multimedia Appendix 1).

### Recruitment

We will recruit 20 to 40 families and their associated or linked health care professionals (eg, allied health professionals and health visitors). There will not be separate recruitment of health care professionals; the only included health care professionals will be those whose clients are also using the app in this study. A central objective of this study is to reach demographic saturation (ethnicity, social-economic background, and education) for family study participants; study recruitment will continue until there is a representative sample from each category. Considering issues related to digital access with regard to socioeconomic status and the impact on information technology use, study recruitment will be regionally focused in a low socioeconomic status area. Recruitment material will be distributed via web-based advertising (Google AdWords), with full study details shared to potential participants via a University of Oxford study recruitment webpage. Once identified, potential participants will be sent copies of all consent and study information (Multimedia Appendix 2 and Multimedia Appendix 3). Participants will then have an opportunity to review the material. If they wish to proceed, they can register for the study.

### Research Participants

Research participants will be limited to parents or legal guardians and their health care professionals using the app. An Amazon gift voucher will be given as compensation to participants who complete the study.

#### Inclusion Criteria

The inclusion criteria are centered on adult users (over 18 years) of the app to focus on interactions associated with family goal setting. Children are excluded from the study to focus on the capability of the app to influence adult participants.

The following inclusion criteria will be used: (1) fluency in English; (2) willingness to use the app; (3) parents or legal guardians of a child or children; (4) health care professionals linked to the parent or guardian; and (5) owner of a smartphone to access the app, with 4G data access.

#### Exclusion Criteria

The following exclusion criteria will be used: (1) individuals who are known to the researchers or staff at Health Education England; (2) deaf or hearing impairment (there is no capability to manage this participant type in this study); (3) prior use of the app before study commencement; (4) refusal to give informed consent; and (5) children, vulnerable young people, or vulnerable adults.

### Study Duration and Follow-Up

The study will last for 9 months (3-month intervention period and 6-month follow-up). Participants will be invited to use the NoObesity app for goal setting and monitoring family activities. The app will be used over the internet via their smartphones at any location during the study period of 3 months. Following a process of informed consent, if participants proceed with the study, they will be given access to links to download and install the app. It is important to note that there are two separate apps, one for families and another for allied health professionals. While the family app is used for family goal planning, the professional app is for monitoring. If health care professionals opt into the study, they will also be interviewed, but separately from the family participants. Interview questions will be asked within the context of how the health care professionals interpreted the impact of the app on their associated client families (same questions will be used but from the perspective of app use and monitoring of outcomes).

### Theoretical Framework

The evaluations of feasibility, acceptability, and usability will be conducted using the following scales and theoretical models or frameworks: (1) system usability scale [12]; (2) the Reach Effectiveness Adoption Implementation Maintenance framework [13] will be used to include information regarding target population reach, the potential for solution impact, adoption by target users, implementation consistency, and costs during delivery and maintenance of the intervention; (3) the Bandura model of health promotion [14] described by social cognitive theory will be used in measurement development and validity, with specific emphasis on self-efficacy, perceived benefits, and perceived barriers [15]; and (4) the Nonadoption, Abandonment and Challenges to the Scale-up, Spread and Suitability framework will be used to evaluate long-term adoption and suitability to further trials [16].

### Data Collection

#### Quantitative Data

App use will be captured for net system use patterns; this will include an examination of app usage and engagement throughout the system. The primary method of evaluating factors impacting uptake will be drawn from qualitative investigation, and data concerning system use will be used as a means to triangulate qualitative findings.

#### Qualitative Data

Surveys and semistructured interviews will be undertaken to evaluate the acceptability and usability of the app. Interviews will be scheduled to last between 40 and 60 minutes (Multimedia Appendix 4). The interviews will be executed at 3-month and 6-month intervals to allow for analysis of the impact of the intervention. Interviews will be conducted through web-based conferencing and telephone conference calls because participants are geographically dispersed. Participants will also be provided with a copy of the study findings that will be published in a peer-reviewed journal.

### Data Analysis

#### Quantitative Data

Web server access logs will be used for analysis of net use patterns (eg, number of screens viewed, number of logins, cumulative minutes using the app, number of plans made, and number of times goals met).

#### Qualitative Data

All interviews will be audio recorded on a digital recorder, transcribed, and coded using thematic analysis [17]. Interviews (at 3-month and 6-month intervals) will be used to explore the factors influencing topic engagement [17] as follows: (1) self-monitoring; (2) goal setting; (3) physical activity and healthy eating support; (4) weight and health assessment; (5) personalized feedback and motivational strategies (rewards, prompts, or gamification); and (6) social support and health care professional involvement.

### Bias

Participants will be asked to provide consent and will be given information on the structure of the study to ensure understanding of the research study. To avoid bias, the criteria to exclude participants who have a relationship with any of the study researchers or who are employed by the university will be enforced. In order to address unconscious bias or other forms of interview recruitment issues, all participants will be included in the study and analysis.

### Risks

Interview questions will avoid areas of culturally sensitive issues and will be purely focused on the impact of the intervention. To control any potential perceived issues in this area, participant confidentiality will be protected using data protection procedures complying with European Union data security legislation.

### Informed Consent

Prior to completing informed consent, participants will be given information that fully describes the process of the study, including why their participation is necessary, how it will be used, and who the results will be reported to. The research team recognizes the rights of the participants to withdraw from the study at any time and have their data destroyed, and participants will be informed of this. If there are issues identified during the study by study participants, they will be documented and escalated to the Head of the Department, who will take action with the principal investigator. The study will also be overseen by a study steering committee who will monitor adherence to the study protocol. Minutes capturing discussions and key points will be recorded and published.

### Data Management

Each participant will be given a unique identifier (ID). The primary key between the unique ID and participant will be held securely on an encrypted secured drive within the university network. The primary key is maintained in the event of a participant wishing to withdraw their data from the study; if such a request is received, all corresponding data and files will be destroyed. Only the research administrators will have access to this file. Although basic demographic information will be captured, the only route to identification will be via this unique ID. Sessions will be recorded and then transcribed by an internal third party (a research assistant trained in transcription) with reference to the unique ID only. The risk of identification will be very low owing to this measure being taken. Only researchers listed in this application and the principal investigator in the research team will have access to the research data. No research data will be transferred to other organizations, and the results will be disseminated via publications. Records of consent will be kept for 3 years after the publication of the final study results. To comply with the General Data Protection Regulation and the Data Protection Act 2018, personal data will be deleted 3 years after the publication of the final study results. All electronic data will be captured and stored on a password-protected network drive within the University of Oxford network. Access to these files will be limited to the principal investigator, the coinvestigator, a research assistant, and a research associate. Electronic data will be coded using the ID and primary critical pseudonymization process.

## Results

This study was funded in March 2019 by Health Education England. A study steering committee was convened in July 2019, where the initial study design was discussed and subsequently reviewed by the principal investigator and the board. After considering feedback, a finalized ethical submission was prepared and approval was received from the University of Oxford Medical Sciences Interdivisional Research Ethics Committee on January 31, 2020 (R62092/RE001). At manuscript submission, study recruitment is pending, and expected results will be published in 2021.

## Discussion

### Overview

This study will provide evidence on the NoObesity app’s influence on self-efficacy and goal setting to improve its design for future studies, if there is evidence of adoption and sustainability. There are gaps in the literature on the lack of effectiveness of mobile apps in improving health behaviors [18] and the need for an iterative design to improve usability [8]. It is hoped that this study will provide variables that can be further evaluated in future studies.

### Methodological Limitations

This study is a limited feasibility study observing factors influencing app usage. It is important to note that this study type does not prove effectiveness.

The study methods have been designed proportionate to the resources available for study execution. A higher investment in study resources could lead to a richer set of results; for example, an iterative approach with families and their children would lead to a richer data set and stronger triangulation of results. Owing to limitations of resources, the study does not have provision for parents or guardians who are deaf or hearing impaired.

The study focuses on the recruitment of participants directly via families; however, direct recruitment of health care professionals could have been an alternate recruitment approach that could lead to more robust targeting of prospective family study participants. This is because health care professionals would have informed views on families having the best potential need for the intervention. The study focuses on family recruitment because of the open nature of access of the app via digital stores and the likelihood that families will access the app promptly. These assumptions will be tested within the study.

There is a risk with regard to the nature of the study. The intervention type will not reach low socioeconomic status demographic participants, and there could be bias toward educated and motivated participants. Use of the technology could potentially create further digital access inequality. An attempt has been made to mitigate this in the study approach through recruitment from a low socioeconomic status geographic area.

The study excludes children as study participants and focuses on parents or guardians. An alternate study design could obtain quantitative or qualitative feedback directly from children.

## Appendix

Multimedia Appendix 1 CONSORT eHEALTH Checklist (V 1.6.1).

Multimedia Appendix 2 Participant consent form.

Multimedia Appendix 3 Information sheet for participants.

Multimedia Appendix 4 Topic guide.

Multimedia Appendix 5 Peer review reports from Oxford University (point by point responses).

## Abbreviations

ID: identifier
