# Supplementary material for: Acceptability and Usability of the Mobile Digital Health App NoObesity for Families and Health Care Professionals: Protocol for a Feasibility Study
Source: JMIR Res Protoc. 2020 Jul 22;9(7):e18068. doi: 10.2196/18068 (PMC7407263; doi:10.2196/18068)
Supplement: Multimedia Appendix 2 [file resprot_v9i7e18068_app2.docx]

##
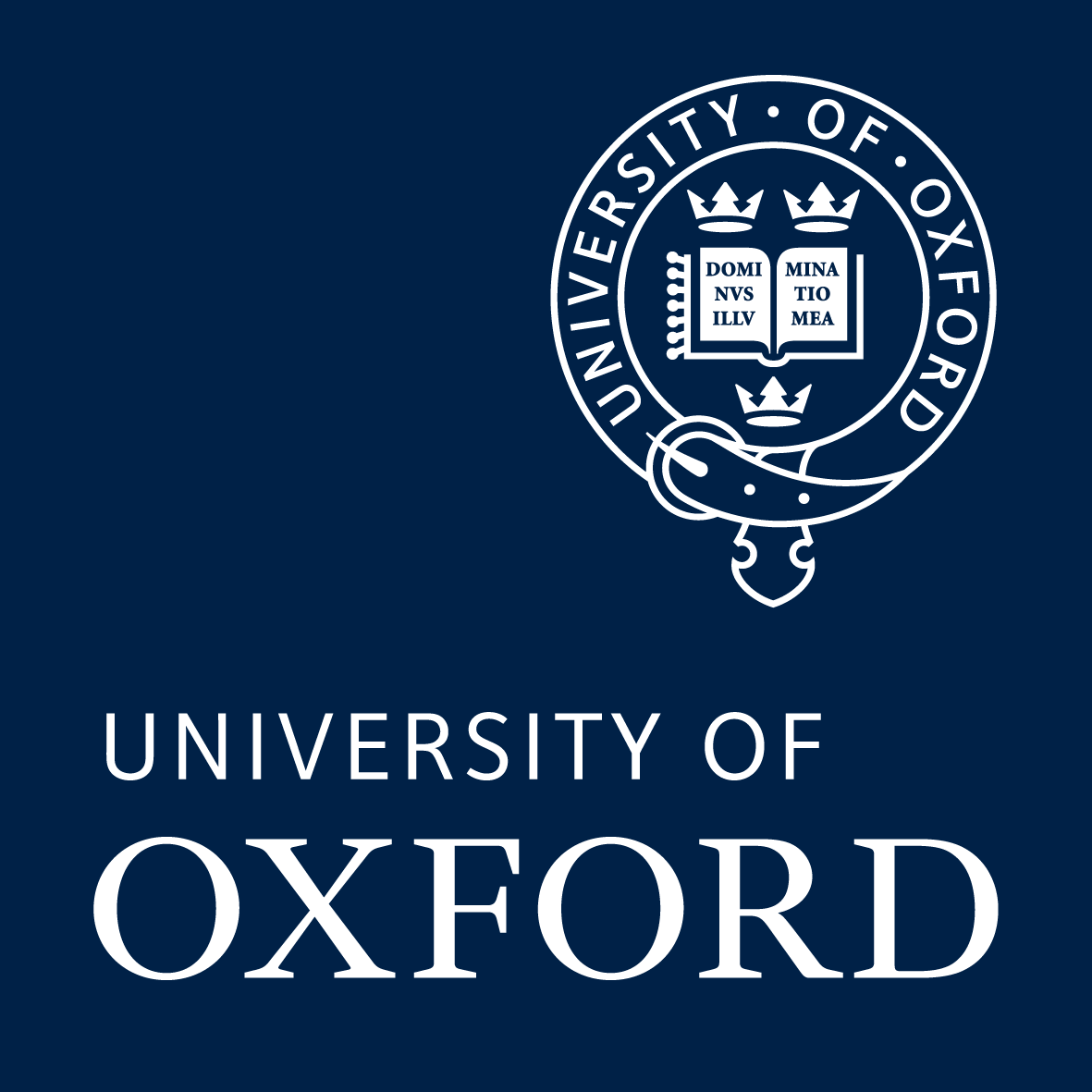
 DEPARTMENT OF PAEDIATRICS

Level 2, Children’s Hospital, John Radcliffe Hospital, Oxford OX3 9DU

EUR ING Dr Edward Meinert, Principal Investigator [edward.meinert@paediatrics.ox.ac.uk](mailto:edward.meinert@paediatrics.ox.ac.uk)

### PARTICIPANT CONSENT FORM

Central University Research Ethics Committee (CUREC) Approval Reference: R62092/RE001

### Feasibility study of the ‘NoObesity’ Digital health App

Purpose of Study: This evaluation will assess Health Education England’s ‘NoObesity’ digital health app’s usability and acceptability to undertake activities improving families’ diet, physical activity and weight. The purpose of the study is to evaluate the app’s influence on self-efficacy and goal setting and to determine what can be learned to improve its design for future studies, should there be evidence of adoption and sustainability.

- 1. I confirm that I have read and understand the information sheet version 3.0 dated September 2019 for the above study. I have had the opportunity to consider the information, ask questions and have had these answered satisfactorily.
  2. I understand that my participation is voluntary and that I am free to withdraw at any time, without giving any reason, and without any adverse consequences or penalty.
  3. I understand that research data collected during the study may be looked at by authorised people outside the research team. I give permission for these individuals to access my data.
  4. I understand that this project has been reviewed by, and received ethics clearance through, the University of Oxford Central University Research Ethics Committee.
  5. I understand who will have access to personal data provided, how the data will be stored and what will happen to the data at the end of the project.
  6. I understand how this research will be written up and published.
  7. I understand how to raise a concern or make a complaint.
  8. I consent to being audio recorded
  9. I understand how audio recordings will be used in research outputs.
  10. I give permission to be quoted directly in research outputs against a pseudonym

*Please initial each box*

- 1. I agree to take part in the study1
  2. **OPTIONAL**

I agree for research data collected in this study to be given to researchers, including those working outside of the EU, to be used in other research studies. I understand that any data that leave the research group will be fully anonymised so that I cannot be identified.

dd / mm / yyyy

#### Name of Participant Date Signature

dd / mm / yyyy

Name of person taking consent Date Signature
