# Supplementary material for: Acceptability and Usability of the Mobile Digital Health App NoObesity for Families and Health Care Professionals: Protocol for a Feasibility Study
Source: JMIR Res Protoc. 2020 Jul 22;9(7):e18068. doi: 10.2196/18068 (PMC7407263; doi:10.2196/18068)
Supplement: Multimedia Appendix 3 [file resprot_v9i7e18068_app3.docx]

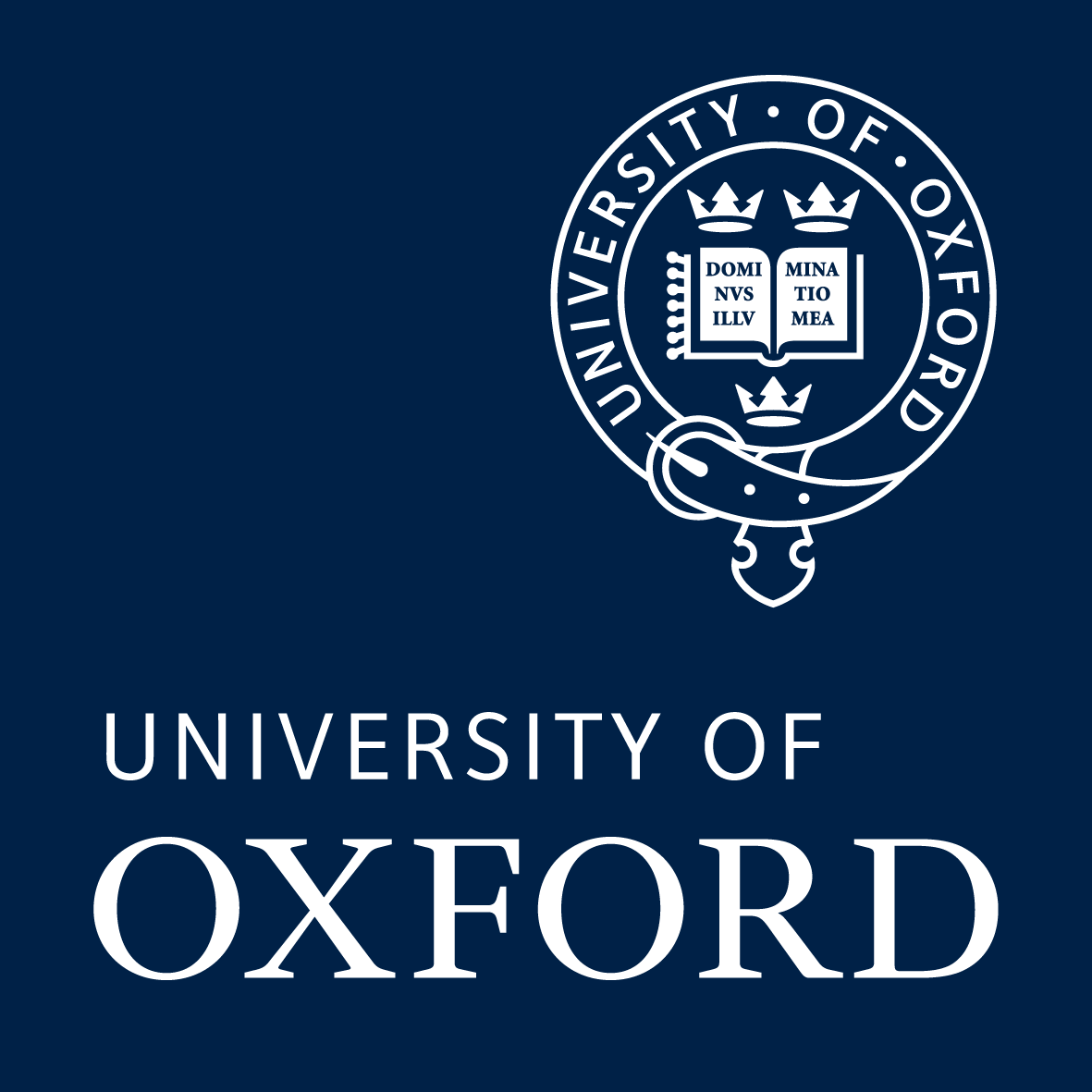
 DEPARTMENT OF PAEDIATRICS

Level 2, Children’s Hospital, John Radcliffe Hospital, Oxford OX3 9DU

EUR ING Dr Edward Meinert, Principal Investigator [edward.meinert@paediatrics.ox.ac.uk](mailto:edward.meinert@paediatrics.ox.ac.uk)

### Feasibility study of the ‘NoObesity’ Digital health App

##### PARTICIPANT INFORMATION SHEET (FAMILY)

Central University Research Ethics Committee (CUREC) Approval Reference: R62092/RE001

We would like to invite you to participate in this research project. You should only participate if you

want to; choosing not to take part will not disadvantage you in any way.

Before you decide whether you want to take part, it is important for you to understand why the

research is being done and what your participation will involve. Please take time to read the

following information carefully and discuss it with others if you wish. Ask us if there is anything that

is not clear or if you would like more information.

###### Why is this research being conducted?

Obesity is a rising concern in the UK, and it is projected that by 2030 41-48% of men and 35-43% of women will be obese. It is estimated that obesity-related conditions are currently costing the NHS £6.1 billion per year, with a cost to society of these conditions estimated at £27 billion per year. A quarter of children aged 0 to 5 in England are overweight or obese and the Department of Health developed a national policy for combating Childhood Obesity in 2018, with critical outcomes of sugar reduction and calorie consumption reduction. Recently, the rapid development of technology has quickly led to a growing market for various devices claiming to aid with weight loss, including the market of wearable digital technology, with 102.4 million wearable devices sold in 2016, and sales expected to continue to rise. Wearable technology refers to any electronics that are worn on the body, commonly being fitness trackers containing some form of an activity monitor and digital health apps on mobile devices used to promote weight management behaviour change. The effectiveness of these technologies has been subject to many studies, with some results suggesting that they can benefit weight loss. However, sustained weight loss is often unsuccessful. While these technologies have significant potential, the limitations noted require further analysis to see if they can be used to make a lasting impact on a positive lifestyle change. This study purpose is to contribute to this evidence base.

This evaluation will assess Health Education England’s NoObesity digital health app’s usability and acceptability to undertake activities improving families’ diet, physical activity and weight. The purpose of the study is to evaluate the app’s influence on self-efficacy and goal setting and to determine what can be learned to improve its design for future studies, should there be evidence of adoption and sustainability.

###### Why have I been invited to take part?

You have been invited because you are an adult who is a parent/legal guardian of a child/children, an owner of a smartphone with data access. Exclusion criteria for this study include researchers known by the researchers or staff at Health Education England and those who have uses of the ‘NoObesity’ app prior to the study commencement.

The study aims involve 16 participants. Should more than 16 indicate interest in partaking, participants will be selected in order to represent as many different working backgrounds as possible.

###### Do I have to take part?

No. You can ask questions about the research before deciding whether or not to take part. If you do agree to take part, you may withdraw yourself from the study at any time, without giving a reason, by advising me

of this decision. Should you decide to withdraw from this study, all data collected regarding your participation shall be deleted within two weeks of notification of study withdrawal.

###### What will happen to me if I take part in the research?

If you are happy to take part in the research, you will be asked to download the ‘NoObesity’ app on your smartphone and use it on average 15 to 30 minutes per day during the study period of three months. The app is designed to provide you with information to track and monitor physical activity, nutrition and other factors impacting measures that could impact your health and wellbeing. The app will be a tool for goal setting and tracking of activities. We will invite your associated allied healthcare professional/health visitor to use a professional version of the app to monitor your progress and participate in this study to provide feedback on the app.

During the research study, we will monitor use of the app, including number of screens viewed, logins, minutes using the app, plans made, and goals met; this activity will be monitored via your interaction with the web services to the application. Following three months of app usage, you will participate in two Skype audio conference or telephone conference for 40 to 60 minutes where you will be asked questions about your experience with using the app; these interviews will be held privately between you and a researcher. The sessions shall be recorded, subject to your permission, and transcribed by a 3rd party at the University of Oxford. If you are a member of NHS staff, you will require line management approval to participate in this study.

If you are still happy to take part, I will ask you to sign a consent form via a digital web page.

###### Are there any potential risks in taking part?

There is a risk that participants may be impacted negatively in terms of the cost to their time that participation would require, therefore the interviews will be completed in less than 60 minutes. The interviews will be transcribed by an internal third party in Imperial and information will not be disclosed externally.

To reduce any potential risks, we shall exclude participants who are known to the research team and seek demographic saturation of participants (bias), ensure confidentially of data collected which may be cultural or psychologically sensitive (sensitivity issues), limit interviews to 60 minutes max to mitigate time loss of participants (time to participate).

1. ***Are there any benefits in taking part?***

By taking part in the study, you will allow the research team to better understand the factors which influence the uptake and use of digital health mobile apps and make recommendations on how to improve this technology based on the study evidence in subsequent iterations.

###### Expenses and payments

You will receive a £100 Amazon gift voucher for compensation for your time.

###### What happens to the data provided?

The information you provide during the study is the **research data**. Any research data from which you can be identified (name, app usage statistics and interview audio recording) is known as **personal data**. This data will be accessed via your digital interactions with the HEE web service via the mobile app and stored for 3 years on a secure university network drive. Audio recordings will be created from interview sessions via a digital recorder. This data will be directly transferred from the device to a secured drive and transcribed by a 3^rd^ party. Once the transcription is completed, the original files will be destroyed.

**Personal / sensitive data** will be stored on a password-protected network drive within the University network. Access to these files will be limited to the PI, the Co-Investigator, a Research Assistant and Research Associate. Electronic data shall be coded using a unique participant number and primary critical pseudonymisation (creation of a fictious name linked to participant numbers) process. Physical copies of consent forms are stored in a locked folder at my office in the John Radcliffe Hospital.

**Other research data** (including consent forms) will be stored for 3 years after publication or public release of the work of the research and stored on secure university network drive. EUR ING Dr Meinert and his research team will have access to the research data. Responsible members of the University of Oxford may be given access to data for monitoring and/or audit of the research.

I would like your permission to use direct quotes against a fictitious name in any research outputs.

I would like your permission to use anonymised data in future studies, and to share data with other researchers. All personal information that could identify you will be removed or changed before information is shared with other researchers or results are made public.

If you consent to take part in the research, any information you provide may be inspected and used by administrators of the study. Each participant will be anonymised using a unique identifier to maintain confidentiality and all data will be securely stored and managed according to University of Oxford rules and expected practices. Raw, un-anonymised audio data will be securely stored separately from the anonymisation key and deleted when it is no longer needed. The anonymised transcripts will be securely stored according to University of Oxford protocols and regulations.

###### Will the research be published?

The research may be published in peer reviewed academic journals.

###### Who is funding the research?

This research is funded by Health Education England. Health Education England has given the University of Oxford unrestricted rights to publish findings of this study.

###### Who has reviewed this study?

This study has been reviewed by, and received ethics clearance through, the University of Oxford Central University Research Ethics Committee (Reference number: R62092/RE001).

###### Who do I contact if I have a concern about the study or I wish to complain?

If you have a concern about any aspect of this study, please contact:

EUR ING Dr Edward Meinert at [edward.meinert@paediatrics.ox.ac.uk](mailto:edward.meinert@paediatrics.ox.ac.uk)

and I will do our best to answer your query. I will acknowledge your concern within 10 working days and give you an indication of how it will be dealt with. If you remain unhappy or wish to make a formal complaint, please contact the Chair of the Research Ethics Committee at the University of Oxford who will seek to resolve the matter as soon as possible:

Chair, **Medical Sciences Inter-Divisional Research Ethics Committee**; Email: ethics@medsci.ox.ac.uk; Address: Research Services, University of Oxford, Wellington Square, Oxford OX1 2JD

###### Data Protection

The University of Oxford is the data controller with respect to your personal data, and as such will determine how your personal data is used in the study.

The University will process your personal data for the purpose of the research outlined above. Research is a task that is performed in the public interest.

Further information about your rights with respect to your personal data is available from [http://www.admin.ox.ac.uk/councilsec/compliance/gdpr/individualrights/.](http://www.admin.ox.ac.uk/councilsec/compliance/gdpr/individualrights/)

###### Further Information and Contact Details

If you would like to discuss the research with someone beforehand (or if you have questions afterwards), please contact:

EUR ING Dr Edward Meinert Department of Paediatrics

Level 2, Children’s Hospital, John Radcliffe Hospital, Oxford OX3 9DU

University email: [edward.meinert@paediatrics.ox.ac.uk](mailto:edward.meinert@paediatrics.ox.ac.uk)


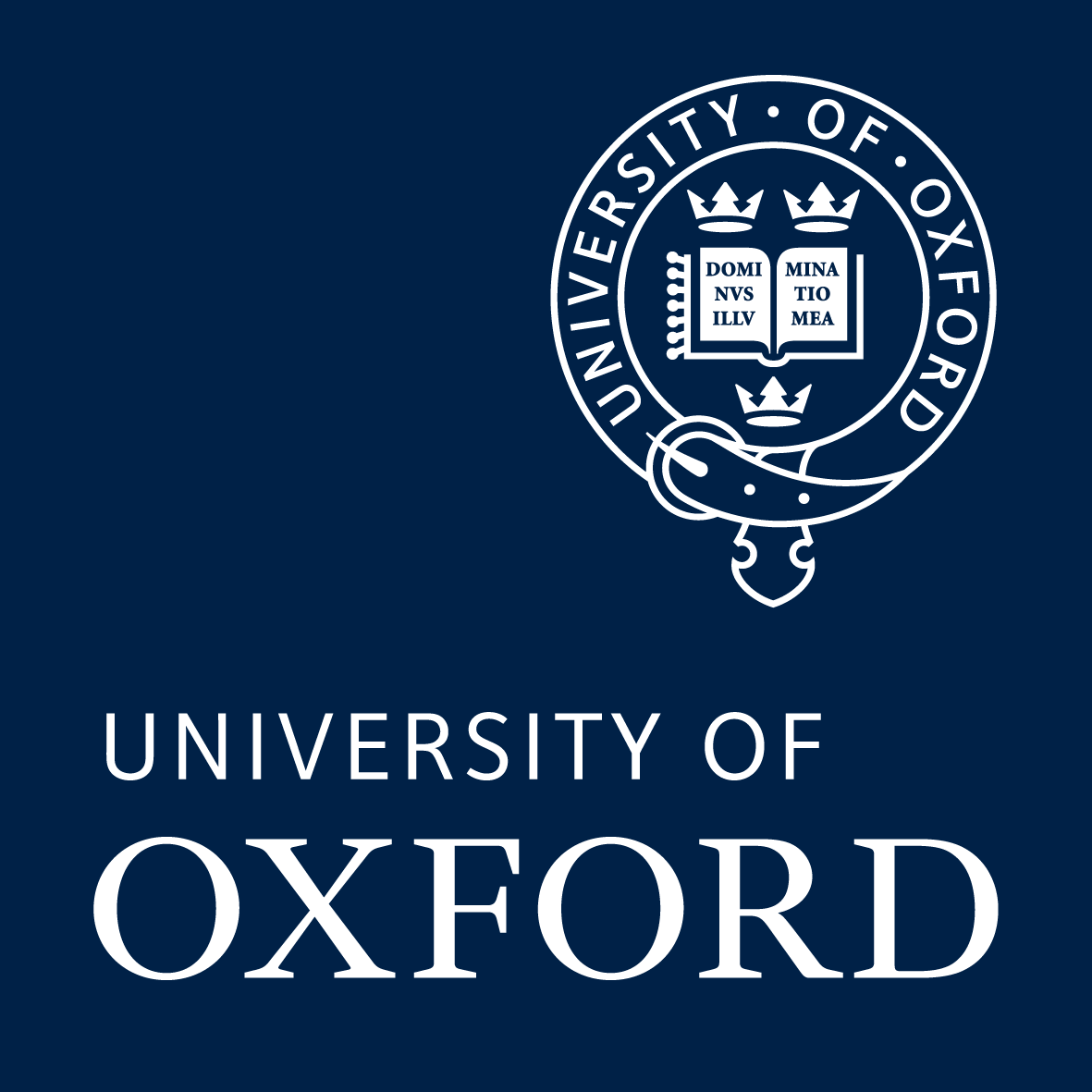
DEPARTMENT OF PAEDIATRICS

Level 2, Children’s Hospital, John Radcliffe Hospital, Oxford OX3 9DU

EUR ING Dr Edward Meinert, Principal Investigator [edward.meinert@paediatrics.ox.ac.uk](mailto:edward.meinert@paediatrics.ox.ac.uk)

### Feasibility study of the ‘NoObesity’ Digital health App

##### PARTICIPANT INFORMATION SHEET (HEALTH CARE / ALLIED HEALTH PROFESSIONAL)

Central University Research Ethics Committee (CUREC) Approval Reference: R62092/RE001

We would like to invite you to participate in this research project. You should only participate if you

want to; choosing not to take part will not disadvantage you in any way.

Before you decide whether you want to take part, it is important for you to understand why the

research is being done and what your participation will involve. Please take time to read the

following information carefully and discuss it with others if you wish. Ask us if there is anything that

is not clear or if you would like more information.

###### Why is this research being conducted?

Obesity is a rising concern in the UK, and it is projected that by 2030 41-48% of men and 35-43% of women will be obese. It is estimated that obesity-related conditions are currently costing the NHS £6.1 billion per year, with a cost to society of these conditions estimated at £27 billion per year. A quarter of children aged 0 to 5 in England are overweight or obese and the Department of Health developed a national policy for combating Childhood Obesity in 2018, with critical outcomes of sugar reduction and calorie consumption reduction. Recently, the rapid development of technology has quickly led to a growing market for various devices claiming to aid with weight loss, including the market of wearable digital technology, with 102.4 million wearable devices sold in 2016, and sales expected to continue to rise. Wearable technology refers to any electronics that are worn on the body, commonly being fitness trackers containing some form of an activity monitor and digital health apps on mobile devices used to promote weight management behaviour change. The effectiveness of these technologies has been subject to many studies, with some results suggesting that they can benefit weight loss. However, sustained weight loss is often unsuccessful. While these technologies have significant potential, the limitations noted require further analysis to see if they can be used to make a lasting impact on a positive lifestyle change. This study purpose is to contribute to this evidence base.

This evaluation will assess Health Education England’s NoObesity digital health app’s usability and acceptability to undertake activities improving families’ diet, physical activity and weight. The purpose of the study is to evaluate the app’s influence on self-efficacy and goal setting and to determine what can be learned to improve its design for future studies, should there be evidence of adoption and sustainability.

###### Why have I been invited to take part?

You have been invited because you are a health care / allied health professional aligned to family who is participating in this research study using the ‘NoObesity’ app. In addition to the family app, there is a Health Care / Allied Health Professional version of the app designed for family monitoring. Exclusion criteria for this study include people known by the researchers or staff at Health Education England and those who have uses of the ‘NoObesity’ app prior to the study commencement.

The study aims involve 16 participants. Should more than 16 indicate interest in partaking, participants will be selected in order to represent as many different working backgrounds as possible.

###### Do I have to take part?

No. You can ask questions about the research before deciding whether or not to take part. If you do agree to take part, you may withdraw yourself from the study at any time, without giving a reason, by advising me

of this decision. Should you decide to withdraw from this study, all data collected regarding your participation shall be deleted within two weeks of notification of study withdrawal.

###### What will happen to me if I take part in the research?

If you are happy to take part in the research, you will be asked to download the ‘NoObesity’ app on your smartphone and use it on average 15 to 30 minutes per day during the study period of three months. You will link the app to your associated family you advise on health. The app will provide you with information to track and monitor physical activity of your patient families, nutrition and other factors impacting measures that could impact your patient families’ health and wellbeing.

During the research study, we will monitor use of the app, including number of screens viewed, logins, minutes using the app, plans made, and goals met; this activity will be monitored via your interaction with the web services to the application. Following three months of app usage, you will participate in two Skype audio conference or telephone conference for 40 to 60 minutes where you will be asked questions about your experience with using the app; these interviews will be held privately between you and a researcher. The sessions shall be recorded, subject to your permission, and transcribed by a 3rd party at the University of Oxford. If you are a member of NHS staff, you will require line management approval to participate in this study.

If you are still happy to take part, I will ask you to sign a consent form via a digital web page.

###### Are there any potential risks in taking part?

There is a risk that participants may be impacted negatively in terms of the cost to their time that participation would require, therefore the interviews will be completed in less than 60 minutes. The interviews will be transcribed by an internal third party in Imperial and information will not be disclosed externally.

To reduce any potential risks, we shall exclude participants who are known to the research team and seek demographic saturation of participants (bias), ensure confidentially of data collected which may be cultural or psychologically sensitive (sensitivity issues), limit interviews to 60 minutes max to mitigate time loss of participants (time to participate).

1. ***Are there any benefits in taking part?***

By taking part in the study, you will allow the research team to better understand the factors which influence the uptake and use of digital health mobile apps and make recommendations on how to improve this technology based on the study evidence in subsequent iterations.

###### Expenses and payments

You will receive a £100 Amazon gift voucher for compensation for your time.

###### What happens to the data provided?

The information you provide during the study is the **research data**. Any research data from which you can be identified (name, app usage statistics and interview audio recording) is known as **personal data**. This data will be accessed via your digital interactions with the HEE web service via the mobile app and stored for 3 years on a secure university network drive. Audio recordings will be created from interview sessions via a digital recorder. This data will be directly transferred from the device to a secured drive and transcribed by a 3^rd^ party. Once the transcription is completed, the original files will be destroyed.

**Personal / sensitive data** will be stored on a password-protected network drive within the University network. Access to these files will be limited to the PI, the Co-Investigator, a Research Assistant and Research Associate. Electronic data shall be coded using a unique participant number and primary critical pseudonymisation (creation of a fictious name linked to participant numbers) process. Physical copies of consent forms are stored in a locked folder at my office in the John Radcliffe Hospital.

**Other research data** (including consent forms) will be stored for 3 years after publication or public release of the work of the research and stored on secure university network drive. EUR ING Dr Meinert and his research team will have access to the research data. Responsible members of the University of Oxford may be given access to data for monitoring and/or audit of the research.

I would like your permission to use direct quotes against a fictitious name in any research outputs.

I would like your permission to use anonymised data in future studies, and to share data with other researchers. All personal information that could identify you will be removed or changed before information is shared with other researchers or results are made public.

If you consent to take part in the research, any information you provide may be inspected and used by administrators of the study. Each participant will be anonymised using a unique identifier to maintain confidentiality and all data will be securely stored and managed according to University of Oxford rules and expected practices. Raw, un-anonymised audio data will be securely stored separately from the anonymisation key and deleted when it is no longer needed. The anonymised transcripts will be securely stored according to University of Oxford protocols and regulations.

###### Will the research be published?

The research may be published in peer reviewed academic journals.

###### Who is funding the research?

This research is funded by Health Education England. Health Education England has given the University of Oxford unrestricted rights to publish findings of this study.

###### Who has reviewed this study?

This study has been reviewed by, and received ethics clearance through, the University of Oxford Central University Research Ethics Committee (Reference number: R62092/RE001).

###### Who do I contact if I have a concern about the study or I wish to complain?

If you have a concern about any aspect of this study, please contact:

EUR ING Dr Edward Meinert at [edward.meinert@paediatrics.ox.ac.uk](mailto:edward.meinert@paediatrics.ox.ac.uk)

and I will do our best to answer your query. I will acknowledge your concern within 10 working days and give you an indication of how it will be dealt with. If you remain unhappy or wish to make a formal complaint, please contact the Chair of the Research Ethics Committee at the University of Oxford who will seek to resolve the matter as soon as possible:

Chair, **Medical Sciences Inter-Divisional Research Ethics Committee**; Email: ethics@medsci.ox.ac.uk; Address: Research Services, University of Oxford, Wellington Square, Oxford OX1 2JD

###### Data Protection

The University of Oxford is the data controller with respect to your personal data, and as such will determine how your personal data is used in the study.

The University will process your personal data for the purpose of the research outlined above. Research is a task that is performed in the public interest.

Further information about your rights with respect to your personal data is available from [http://www.admin.ox.ac.uk/councilsec/compliance/gdpr/individualrights/.](http://www.admin.ox.ac.uk/councilsec/compliance/gdpr/individualrights/)

###### Further Information and Contact Details

If you would like to discuss the research with someone beforehand (or if you have questions afterwards), please contact:

EUR ING Dr Edward Meinert Department of Paediatrics

Level 2, Children’s Hospital, John Radcliffe Hospital, Oxford OX3 9DU

University email: [edward.meinert@paediatrics.ox.ac.uk](mailto:edward.meinert@paediatrics.ox.ac.uk)
