# Supplementary material for: Acceptability and Usability of the Mobile Digital Health App NoObesity for Families and Health Care Professionals: Protocol for a Feasibility Study
Source: JMIR Res Protoc. 2020 Jul 22;9(7):e18068. doi: 10.2196/18068 (PMC7407263; doi:10.2196/18068)
Supplement: Multimedia Appendix 4 [file resprot_v9i7e18068_app4.docx]

# TOPIC GUIDE

**Title of Project: Feasibility study of the ‘NoObesity’ Digital Health App**

**Name of researcher:** Dr Edward Meinert Research questions:

- *What issues influence self-efficacy, perceived benefits and barriers for digitally delivered interventions to prevent childhood obesity?*
- *How can interventions delivered digitally build communication among participants (parents/guardians and health care professionals) to create engagement and education on positive lifestyle choices for families?*

Interview questions:

- **Participant background (please note the first seven questions are optional)**

1. What gender do you identify as?
2. What is your age?
3. Please specify your ethnicity?
4. What town do you live in?
5. What is the highest degree or level of education you have completed?
6. What is your annual household income?
7. What is your current employment status?
8. Can you describe your family for me?
9. Do you have any experience using apps on your smartphone for healthy living?

- **General app usability and acceptability**

1. How has your and your family’s experience using the ‘NoObesity’ app been?
2. What has happened as a result of using the app in your family?

- **Self-monitoring and goal setting**

1. What do you think of the app function that helps to monitor your family’s habits?
2. Did the app help you set goals? How come?

- **Physical activity and healthy eating support**

1. How did the app support your family to exercise and eat healthy? Why (not)?

- **Weight and health assessment**

1. What was your experience with the app’s weight and health measurements?

- **Personalised feedback and motivational strategies (rewards, prompts)**

1. What did you think of the app’s feedback?
2. Did the app motivate your family? What helped or didn’t help? How come?

- **Social support and health care expert involvement**

1. Were you in touch with other parents/guardians through the app? How?
2. Can you describe how your health care worker was involved in using the app?

- **Feedback**

1. What would you suggest to make the app better?
2. Is there anything else you’d like to mention?
