# Supplementary material for: Acceptability and Usability of the Mobile Digital Health App NoObesity for Families and Health Care Professionals: Protocol for a Feasibility Study
Source: JMIR Res Protoc. 2020 Jul 22;9(7):e18068. doi: 10.2196/18068 (PMC7407263; doi:10.2196/18068)
Supplement: Multimedia Appendix 5 [file resprot_v9i7e18068_app5.docx]

| JMIR Research Protocols |  |  |
| --- | --- | --- |

25 April 2020

Dear Editor,

Thank you for your consideration of our manuscript “Usability of the mobile digital health ‘NoObesity’ app for families and healthcare professionals: a feasibility study” for publication in JMIR Research Protocols. We have made amendments to the manuscript, reflecting the valuable review comments forwarded and feel the paper has been well enhanced to be re-presented for publication. Please let us know if there is anything we can further do to clarify or extend our paper.

With kind regards,

Edward Meinert

Enclosures:

1. Chart mapping peer review feedback

| **ID** | **Reviewer** | **Reviewer Feedback** | **Author response** | **Action taken** |
| --- | --- | --- | --- | --- |
| 1 | O | This is a protocol for a mixed method research. The research design is reasonable, but the statistical analysis needs to be refined, that is, to improve the specific measures of statistical analysis, including qualitative and quantitative data. | We thank the reviewer for their time and consideration of this research protocol. Statistical analysis will be vital when looking at evaluation of effectiveness of the solution, however at this stage of research which is concerned on usability, the primary method of evaluation is examining factors impacting uptake and use and therefore the investigation is primarily qualitative and as such, statistical and quantitative methods are not in play. There will be some work done on quantitative analysis of use trends within the solution, but the methods for this work are a primary outcome of the study. | Extended quantitative data narrative to include further details of study focus. |
| 2 | O | In addition, it is strange that there are no references in the discussion section. Please add appropriate references to the discussion. | There is a lack of evidence on the impact of mobile apps on influence weight management; it is hoped this study will provide data to improve understanding. | Discussion amended |
| 3 | AC | This paper is about the development and a preliminary test of NoObesity APP for families and healthcare professionals. Personally I think this is very innovative and a great idea and that it can be very useful not only in hospital but also at home. I encourage the authors to continue the work with this tool in order to improve it and being able to release it for clinical practice and Home Test. However, there are some objections I have found in the document. | We thank the reviewer for their time and consideration of this research protocol. | N/A |
| 4 | AC | In this study, the authors have demonstrated good evaluation outcomes to our potential readers. Yet, it is advised that the authors describe more about the NoObestiy APP. | We have included additional data to describe the app functionality in this revision. | Amended background to include details to the app implementation. |
| 5 | AC | The authors should include more information about how the inclusion criteria and exclusion criteria was designed and how the protocol was applied. | The inclusion criteria centred on adult users of the apps to focus on interactions associated with family goal-setting. Children were excluded from the study to focus on the capability of the app to influence adult participants. | Inclusion criteria amended. |
| 6 | AC | The study methods are appropriately designed. Overall, the topic is interesting and innovative. It could be a good idea to state clearly in the Discussion section the limitations of this study in order to make it clear that there are needed more tests to release the tool for clinical use. | We thank the reviewer for the comment on design. We have made a slight amendment to the limitations section to indicate the need for more tests. | Limitations amended. |
| 7 | AC | The author did not declare the NoObestiy APP. Furthermore, I am afraid the privacy policy of the APP | The COIs have been more clearly stated in the COI section. | COIs revised. |
| 8 | AR | It is a very interesting project, I hope it can be carried out and thus address a problem as serious as obesity today. However, there are several points to be resolved in the submitted manuscript. | We thank the reviewer for their time and consideration of this research protocol. | N/A |
| 9 | AR | It gives the impression at the beginning of the reading that the application will be aimed at children. The wording should be improved since according to the title it is for all family members (and the problem of obesity is too). | We have extensively modified the manuscript to detail the system use case and the study inclusion criteria. | N/A |
| 10 | AR | Remove this quote from the summary "UK Department of Health and Social Care: Global Public Health Directorate: Obesity, Food and Nutrition / 10800: Childhood obesity: a plan for action - Chapter 2)" | Removed | Manuscript updated. |
| 11 | AR | There are repeated words in the text (check the wording). | Grammar/style will be corrected in copyediting, though we have made grammar modifications in the manuscript. | Manuscript updated. |
| 12 | AR | In the first paragraph of the introduction, the information "(UK Department of Health and Social Care: Global Public Health Directorate: Obesity, Food and Nutrition / 10800: Childhood obesity: a plan for action - Chapter 2)" should be handled by cited and reference. | Citation added | Manuscript updated. |
| 13 | AR | In the introduction in the second paragraph. What bibliography supports these statements? "with some results suggesting that they can benefit weight loss temporarily. However, sustained weight loss is often unsuccessful. Even where evidence on these interventions demonstrate a significant weight loss or reduction in weight gain, if this benefit only lasts for a short period of time, then this dramatically reduces the usefulness of these technologies." | This paragraph has several citations which source where this information is sourced from including systematic reviews completed by the authors. | N/A |
| 14 | AR | In the third paragraph of the introduction, "The user testing consisted of research with professionals and parents / carers which was conducted via focus groups. [11]." Do not cite a web page with user testing, it would enrich this protocol more if you describe how those tests were, and what you got from those tests. | We have amended the manuscript to include a solution overview section which details system functions. | Manuscript amended. |
| 15 | AR | Is it possible that one of the risks is the abandonment of study participants? | This is a good point, but this is accounted for in the study design. Abandonment of participants is one of the issues we wish to test in the study. | N/A |
| 16 | AR | Citations in the text are being made incorrectly, for example in the first paragraph of the introduction collate [2,3,4], the correct form is [2-4] because the numbering of citations is continuous. Also in paragraph two [6,7,8,9,10], the correct form is [6-10], they should review the Vancouver Standards throughout the document. | This is noted; this will be corrected in copyediting. | N/A |
| 17 | AR | References do not follow the Vancouver format, I strongly recommend using a reference manager for references as for appointments. | This is noted; this will be corrected in copyediting. | N/A |
| 18 | AR | Whenever there is a scientific article, the article should be cited and not a web page (Case of reference 12) | This is noted; this will be corrected in copyediting. | N/A |
| 19 | AS | The research question should belong to the introduction section. | The RQs have been moved as per the feedback. | Introduction amended. |
| 20 | AS | The research protocol was too detailed. I do not think this fulfills the journal's space and formatting. Authors may want to take some time and modify the content more for the journal publication. | This is noted; this will be corrected in copyediting. | N/A |
| 21 | AS | The authors' information was not clear as I did not see 3rd-10th organizations matched with any author. | The COIs have been more clearly stated in the COI section. | COIs revised. |
| 22 | AS | No citation should appear in the abstract. | The abstract has been amended to reflect this feedback. | Abstract amended |
| 23 | AS | Keywords: Mhealth and mobile health are the same | This is true but may be reflected separately in search results. | N/A |
| 24 | AS | It is projected? that by 2030 41-48% | This is correct. | N/A |
| 25 | AS | Only researchers listed in this application? and the PI’s in the research team will have access to research data. | The data policy is listed in the manuscript Author affiliations have been corrected. | The abstract has been modified. |
